# Phase 1 study of AYP-101 (soybean phosphatidylcholine): safety, pharmacokinetics, and lipid profile effects for reducing submental fat

**DOI:** 10.1186/s12944-024-02387-4

**Published:** 2024-12-28

**Authors:** Hyun Joon Lee, Xu Jiang, A. M. Abd El-Aty, Ji Hoon Jeong, Jae-Yong Chung

**Affiliations:** 1 Department of Clinical Pharmacology and Therapeutics, Seoul National University College of Medicine and Bundang Hospital, Seongnam, 13620 Republic of Korea; 2 Department of Health Science and Technology, Seoul National University College of Convergence Science and Technology, Seongnam, 13620 Republic of Korea; 3 Department of Pharmacology, College of Medicine, Yonsei University, Seoul, 03722 Republic of Korea; 4 Department of Pharmacology, Faculty of Veterinary Medicine, Cairo University, Giza, 12211 Egypt; 5 Department of Medical Pharmacology, Medical Faculty, Ataturk University, Erzurum, 25240 Turkey; 6 Department of Pharmacology, College of Medicine, Chung-Ang University, Seoul, 06974 Republic of Korea; 7 Department of Global Innovative Drugs, Graduate School of Chung-Ang University, Seoul, 06974 Republic of Korea

**Keywords:** Soybean phosphatidylcholine, Safety, Pharmacokinetics, Lipid

## Abstract

**Background:**

Excessive submental fat under the chin is a known aesthetic concern because of its negative impact on facial appearance and psychological well-being. AYP-101 is a newly developed injectable agent containing 93% soybean phosphatidylcholine (SPC) designed to reduce submental fat. We conducted a phase 1 study to evaluate the safety, pharmacokinetic (PK), and lipid profile effects of AYP-101.

**Methods:**

This study was a randomized, double-blind, placebo-controlled, single-dose, two-cohort trial. Participants were randomized into groups receiving 250 mg of AYP-101, 500 mg of AYP-101, or placebo at a 9:9:8 ratio across both cohorts. Safety was assessed through standard clinical evaluations. Blood samples were collected up to 144 h postdose in cohort 1 and up to 48 h postdose in cohort 2. The PK parameters were calculated via noncompartmental analysis.

**Results:**

All 26 randomized subjects completed the study. A total of 72 solicited adverse drug reactions (ADRs) were reported by 24 subjects, all of which were mild. The most common ADRs were injection site pain and bruising. There were 29 ADR cases in the 250 mg group; 26 cases in the 500 mg group; and 18 cases in the placebo group, indicating that there were no clinically significant differences in the safety profiles between the groups. All the dose groups presented similar PK profiles, with C_max_ values of 12.86, 13.44, and 13.61 µg/L and AUC_0−24 h_ values of 278.06, 274.79, and 267.63 µg*h/L, respectively. No clinically significant differences in PK or lipid profiles were observed between the postdose group and the baseline group for any of the dose groups.

**Conclusions:**

AYP-101 appears to be a safe candidate for treating submental fat, with localized reactions and no systemic exposure at single subcutaneous doses of up to 500 mg.

**Trial registration:**

ClinicalTrials.gov NCT05476094.

**Supplementary Information:**

The online version contains supplementary material available at 10.1186/s12944-024-02387-4.

## Background

Excessive submental fat (SMF) under the chin is a cosmetic concern because it leads to skin laxity, unattractive facial contours, and an aged appearance. Its presence is often linked to factors such as weight, age, and lifestyle, with an observed correlation between excessive SMF and dissatisfaction with facial appearance, thereby affecting psychological well-being [1–4]. Currently, the management of excessive SMF primarily involves two modalities: surgical interventions, such as liposuction, and nonsurgical approaches involving the use of medical devices or injections [5, 6]. However, both methods have inherent limitations. Surgical interventions may not be universally applicable due to associated risks, such as general anesthesia, bleeding, bacterial infection, and a prolonged recovery period. There is limited clinical evidence supporting the efficacy and safety of SMF removal for nonsurgical methods involving the use of medical devices, such as laser therapy. Furthermore, there is debate surrounding the role of laser treatment for fat removal, with some considering it solely adjunctive to liposuction [3]. Another popular nonsurgical approach involves the injection of a chemical mixture of drugs into the localized fat [7].

Deoxycholic acid (DCA) has previously received global approval for reducing submental fat (SMF) [8]. However, DCA is known to cause nonselective cell necrosis with hyperinflammation, which can lead to clinical symptoms such as pain, edema, and other local adverse events [9–11]. Additionally, severe side effects, such as skin ulceration and nerve damage due to nonselective cell lysis, have been reported [12]. As a result, there is an urgent need for a new, clinically safe drug that offers comparable efficacy for reducing SMF [2, 13]. AYP-101 (AMI Pharm Co., Ltd., Seongnam-si, Gyeonggi-do, Republic of Korea) is a novel treatment for excessive SMF that consists of high-purity soybean phosphatidylcholine (SPC) extracted from soybeans. SPC is an essential phospholipid and esterified glycerophospholipid with fatty acids at positions 1 and 2 and phosphocholine at position 3. In vitro efficacy studies have shown that SPC selectively reduces adipocytes in a time- and concentration-dependent manner, suggesting its potential as a safe and effective treatment for localized SMFs [14]. When administered without a solubilizer, SPC faces challenges related to formulation stability. Although SPC alone has a concentration-dependent effect on the dissolution of adipocytes, its low stability makes it unsuitable for maintaining quality control standards. Therefore, adding a stabilizing agent is necessary to ensure long-term dispersion in water.

Previously, DCA was used as a solubilizer in combination with SPC. However, when injected subcutaneously alone or with SPC, DCA inhibited the selective adipocyte apoptosis and lipolysis of SPC through a nonselective cell lytic action accompanied by hyperinflammation [11]. Consequently, DCA was determined to be an unsuitable solubilizer. Instead of DCA, glycocholic acid (GCA) was selected as the solubilizer for AYP-101 on the basis of previous preclinical studies. These studies indicated that GCA does not induce intrinsic effects on fat cell reduction or elevation and enhances formulation stability. Furthermore, in vitro adipocyte viability efficacy tests using SPC alone and AYP-101, an SPC injection that solubilizes SPC with GCA, revealed no statistically significant difference in adipocyte viability, and the formulation stability was superior to that of SPC alone. AYP-101, a formulation of SPC solubilized in GCA, demonstrated excellent formulation stability without compromising quality control. Moreover, it is considered an effective formulation for selective adipocyte reduction. Because there are no clinical data on AYP-101, we conducted the first-in-human study to evaluate its safety, tolerance, pharmacokinetics (PK), and effects on the lipid profiles of the two groups treated with AYP-101.

## Methods

### Study subjects

Given that this was the first in-human study to confirm the PK properties of the investigational product, it was divided into two cohorts. The number of subjects in each cohort was determined by referencing typical phase 1 clinical trials. Subjects were selected on the basis of screening tests, which included medical history, physical examination, and laboratory tests.

Eligible subjects were healthy adults aged 19–65 years at the time of the screening. The subjects needed to have sufficient subcutaneous fat under the chin to permit a single subcutaneous injection of the investigational product into 50 points, or at least 45 points, at 1.0 cm grid intervals. To ensure that the subjects had sufficient subcutaneous fat, each participant was visually assessed to determine whether the designed grid pad fully covered the injection site (from the mandible to the mentum) during the screening test. Subjects were excluded if they lacked sufficient subcutaneous fat under the chin, had previously undergone cosmetic surgery, or had received injections containing SPC and DCA. Furthermore, subjects were excluded if they had a history of allergy or hypersensitivity to components of the SPC, lidocaine, or any study-related medical devices. Patients with diseases, inflammation, wounds, or surgical scars at the injection site as well as those with a past or current history of dysphagia were also excluded.

Written informed consent was obtained from all the subjects prior to any study-related procedure. The study was approved by the institutional review board of Seoul National University Bundang Hospital and conducted in accordance with the Declaration of Helsinki (Clinical Research Information Service registration no. NCT05476094).

### Study design and drug administration

A randomized, double-blind, placebo-controlled, single-dose, two-cohort study was conducted to evaluate the safety, tolerability, PK, and effects on the lipid profiles of the investigational products. On the basis of the time‒concentration profile observed after administering the investigational products in cohort 1, decisions were made regarding the progression to cohort 2. Adjustments were made to the study protocol for cohort 2, including modifications to the hospitalization period and blood sampling times. In cohort 1, a minimum of eight eligible subjects were planned to be enrolled without accounting for dropout rates. For cohort 2, a minimum of 18 eligible subjects were planned, considering a 20% dropout rate. The subjects were randomly assigned to one of three groups: AYP-101 250 mg, AYP-101 500 mg, or placebo. The selection of these doses was based on ongoing multiple-dose clinical trials and preclinical efficacy studies. The subject allocation ratio was 3:3:2 for cohort 1, with a total of 8 subjects, and 1:1:1 for cohort 2, with a total of 18 subjects. The randomization code was generated via SAS (version 9.4 or higher), with the block size set to a random multiple of the number of treatment groups. Following randomization, the subjects received the investigational product subcutaneously under the chin at each dose level. The randomization codes for each group remained blinded to treatment assignment for each subject until after study completion, data lock, and central laboratory analysis to minimize bias.

The investigational product was administered in a single session with multiple divided injections to cover the submental area. The preparation involved sterilizing pens, alcohol swabs, grid pads for marking injection sites, gauze, tape, and local anesthesia with lidocaine ointment applied to the injection site for at least 30 min. Subjects were briefed on potential solicited adverse events.

**Fig. 1:**
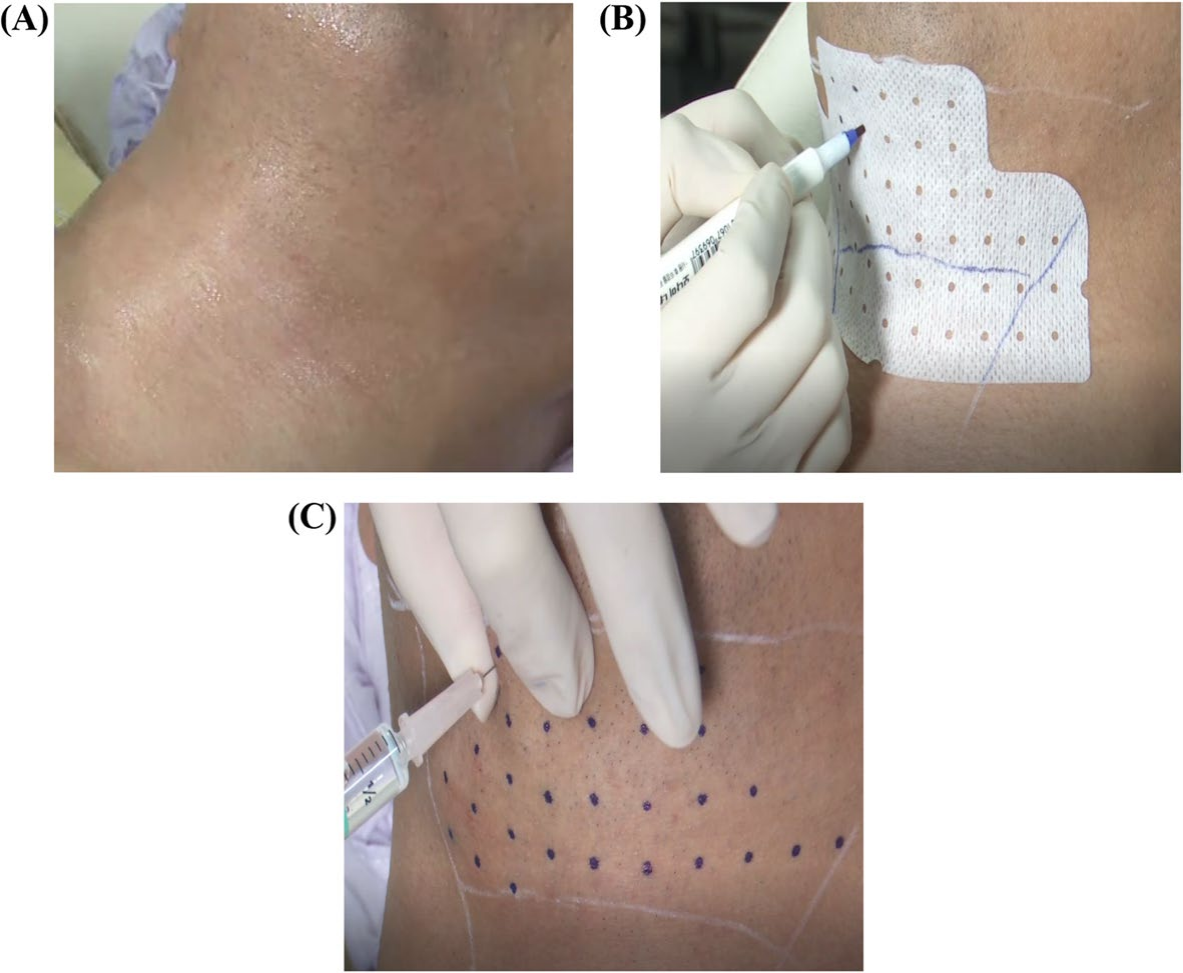
Scheme of the injection procedure. **A **After applying lidocaine ointment, the treated area was sanitized with an alcohol swab. **B **A grid pad, designed with 1.0 cm intervals, was applied from the angle of the mandible down to the lower border of the mentum and the injection sites were marked as dots within the grid. **C **After pinching the fat with two fingers, a total of 10 mL of investigational product (0.2 mL per point, for a total of 50 points, with at least 45 points if insufficient) was injected into the marked site

The subject was administered local anesthesia with a lidocaine ointment for at least 30 min. A grid pad designed to be placed at 1.0 cm intervals from the angle of the mandible down to the lower border of the mentum was attached to the subjects. The injection site was marked with a dot to ensure that the injections were below the inferior border of the mandible. The investigational product was then injected into the broad area immediately adjacent to each marked injection site. The investigational product was then injected into submental fat tissue adjacent to marked sites, totaling 10 mL (0.2 mL per point, for a total of 50 points, with at least 45 points if insufficient) administered at 1.0 cm intervals with 30G needle syringes (Fig. 1).

### Safety and tolerability assessments

Safety and tolerability assessments included monitoring adverse events (AEs), vital signs, physical examinations, 12-lead electrocardiograms, and clinical laboratory tests. AEs were categorized into unsolicited AEs and solicited AEs. To assess solicited and unsolicited adverse events, the subjects were followed up by visiting the clinical trial site at 2, 3, 7, 14, and 28 (final visit) days after administration. Solicited AEs included numbness, erythema, induration, paresthesia, nodules, skin pulling, bruising, discomfort, hematoma, pain, pruritus, swelling, and warmth at the injection site. The confirmation of edema among the AEs at the injection site was based on clinical photographs taken during the study.

### PK assessments

The main active species of SPC, 1,2-dilinoleoyl-sn-glycero-3-phosphocholine (DLPC), was selected as an indicator of essential phospholipids. The concentration of DLPCs in the plasma samples was determined via validated liquid chromatography coupled with tandem mass spectrometry (LC‒MS/MS).

Given that the investigational products were endogenous substances that could be affected by diet, the subjects were admitted at −7 D and provided with a restricted standard diet that did not contain soybeans or egg yolk.

Considering the diurnal variation of PK evaluation, baseline blood PK sampling for DLPC was conducted at 0.5 h (−1D), 1 h (−1D), 1.5 h (−1D), 3 h (−1D), 6 h (−1D), 9 h (−1D), 12 h (−1D), and 24 h (−1D, 1D 0 h) predose. In cohort 1, subsequent PK sampling was conducted at predose (1D, 0 h), 0.5 h (1D), 1 h (1D), 1.5 h (1D), 3 h (1D), 6 h (1D), 9 h (1D), 12 h (1D), 24 h (2D), 48 h (3D), 72 h (4D), 96 (5D), 120 (6D), and 144 (7D) after dosing. In cohort 2, baseline blood PK sampling for DLPCs was conducted at the same time points as in cohort 1, with subsequent PK sampling conducted at predose (1D 0 h), 0.5 h (1D), 1 h (1D), 1.5 h (1D), 3 h (1D), 6 h (1D), 9 h (1D), 12 h (1D), 24 h (2D), and 48 h (3D) after dosing.

The noncompartmental method was performed via appropriate software to calculate the PK parameters of the DLPC. For the PK parameters, the following values were calculated from the DLPC concentration measured: the maximum plasma concentration (C_max_), time to reach C_max_ (T_max_), area under the plasma concentration‒time curve up to 24 h (AUC_0−24 h_), area under the plasma concentration‒time curve extrapolated to infinity (AUC_inf_), clearance (CL), elimination rate constant (λ_z_), volume of distribution (V_d_), terminal half-life (t_1/2_), baseline adjusted C_max_ (ΔC_max_), and AUC_0−24 h_ value (ΔAUC_0−24 h_). The baseline adjusted value was calculated by subtracting the concentration at the baseline blood sampling point from the contrasting postdose blood sampling point. Furthermore, to determine the effects on the lipid profile of the investigational product, the T_max_, C_max_, AUC_0−24 h_, AUC_inf_, ΔC_max_, and ΔAUC_0−24 h_ of the total serum cholesterol, triglyceride, and free fatty acid levels were determined.

### Statistical analyses

Continuous data, including the number of test subjects, mean, standard deviation, coefficient of variation (%), minimum value, median, and maximum value, are presented via descriptive statistics. Categorical data are presented as frequencies (N) and ratios (%). In the context of PK evaluation, the baseline value at each time point before 24 h of 1D was a symmetrical time point of −1D, and the baseline value at the time point after 48 h of 1D was the value measured at 0 h of 1D. Unless otherwise specified, values before the first dose in each period were used. Statistical analyses were performed via SAS^®^ version 9.4 (SAS Institute, Cary, NC).

### Bioanalytical methods

DLPC-d6 was used as an internal standard. The mobile phases consisted of ammonium formate and acetonitrile at a 40:60 ratio under gradient conditions with a flow rate of 0.4 mL/min. The concentration of DLPC in the plasma sample was determined via validated LC‒MS/MS via a Waters ACQUITY UPLC© and a Waters Xevo© instrument. An analytical column (ACQUITY UPLC^®^ Protein BEH C4 300 A, 2.1 × 50 mm, 1.7 μm) was used for chromatographic separations. Mass spectrometry was performed under positive ESI ionization mode (*m/z* for DLPC 782.5 → 184.2, *m/z* for DLPC-d6 788.6 → 184.1).

## Results

### Study subjects and demographics

Across the two cohorts, a total of 26 healthy adult men and women were randomly assigned at a ratio of 9:9:8 to two treatment groups (AYP-101 250 mg and 500 mg) and a placebo group. In cohort 1, 8 subjects were randomized to the treatment group or placebo group at a ratio of 3:3:2, whereas in cohort 2, 18 subjects were randomized at a ratio of 1:1:1. All randomized subjects received the investigational product according to their assigned group and completed the study without any dropouts; all subjects received the investigational product via a complete 50-point injection. Overall, 22 of the subjects were male, and 4 were female. The means ± standard deviations of the age, weight, height, and BMI of the subjects were 28.04 ± 5.40 years, 82.38 ± 13.04 kg, 173.98 ± 7.62 cm, and 27.15 ± 3.69 kg/m^2^, respectively. The demographic data were not significantly different among the three groups (Table S1).

### Safety and tolerability results

All 26 subjects who were administered investigational products were included in the safety analysis. A total of 73 AEs were observed in 24 subjects who received at least one injection of the investigational product. Among the 73 AEs, 72 were solicited AEs, and there was only one unsolicited AE (salivary gland enlargement) in the AYP-101 500 mg treatment group. Except for this AE, all 72 AEs were confirmed to be related to the investigational product and were thus classified as adverse drug reactions (ADRs). Among the 72 ADRs, injection site pain was the most common ADR, occurring in 20 of the 20 cases, followed by injection site bruising, which occurred in 18 of the 18 cases (Table 1).

**Table 1:** Summary of unsolicited adverse events (AEs) and adverse drug reactions (ADRs)

	AYP-101 250 mg (*N* = 9)	AYP-101 500 mg (*N* = 9)	Placebo (*N* = 8)	All Subjects (*N* = 26)
**Unsolicited AEs**	.	1 (11.1)	.	1 (11.1)
Salivary gland enlargement	.	1 (11.1)	.	1 (11.1)
**Subjects with at least one ADRs**	**9 (100.0)**	**9 (100.0)**	**6 (75.0)**	**24 (92.3)**
**General disorders and administration site conditions**	**9 (100.0)**	**9 (100.0)**	**6 (75.0)**	**24 (92.3)**
Bruising	7 (77.8)	7 (77.8)	4 (50.0)	18 (69.2)
Discomfort	7 (77.8)	7 (77.8)	1 (12.5)	15 (57.7)
Hematoma	.	.	1 (12.5)	1 (3.8)
Induration	1 (11.1)	.	1 (12.5)	2 (7.7)
Pain	9 (100.0)	8 (88.9)	3 (37.5)	20 (76.9)
Pruritus	.	.	1 (12.5)	1 (3.8)
Swelling	4 (44.4)	2 (22.2)	5 (62.5)	11 (42.3)
Warmth	1 (11.1)	1 (11.1)	2 (25.0)	4 (15.4)

Data are displayed as number of subjects (percentage of subjects). Percentages are based on the subjects within each treatment group.

In the treatment groups, the most common AE in the AYP-101 250 mg and 500 mg groups was injection site pain, which occurred in 9 of the 9 patients and 8 of the 8 patients, respectively. In the placebo group, injection site bruising was the most common AE and was observed in 4 of the 4 patients.

All the ADRs were mild, and no subjects discontinued or died due to ADRs. There were no unexpected ADRs, and serious adverse events requiring medical attention occurred. There were no significant changes in vital signs, clinical laboratory tests, 12-lead electrocardiograms, or physical examinations.

### PK results

**Fig. 2:**
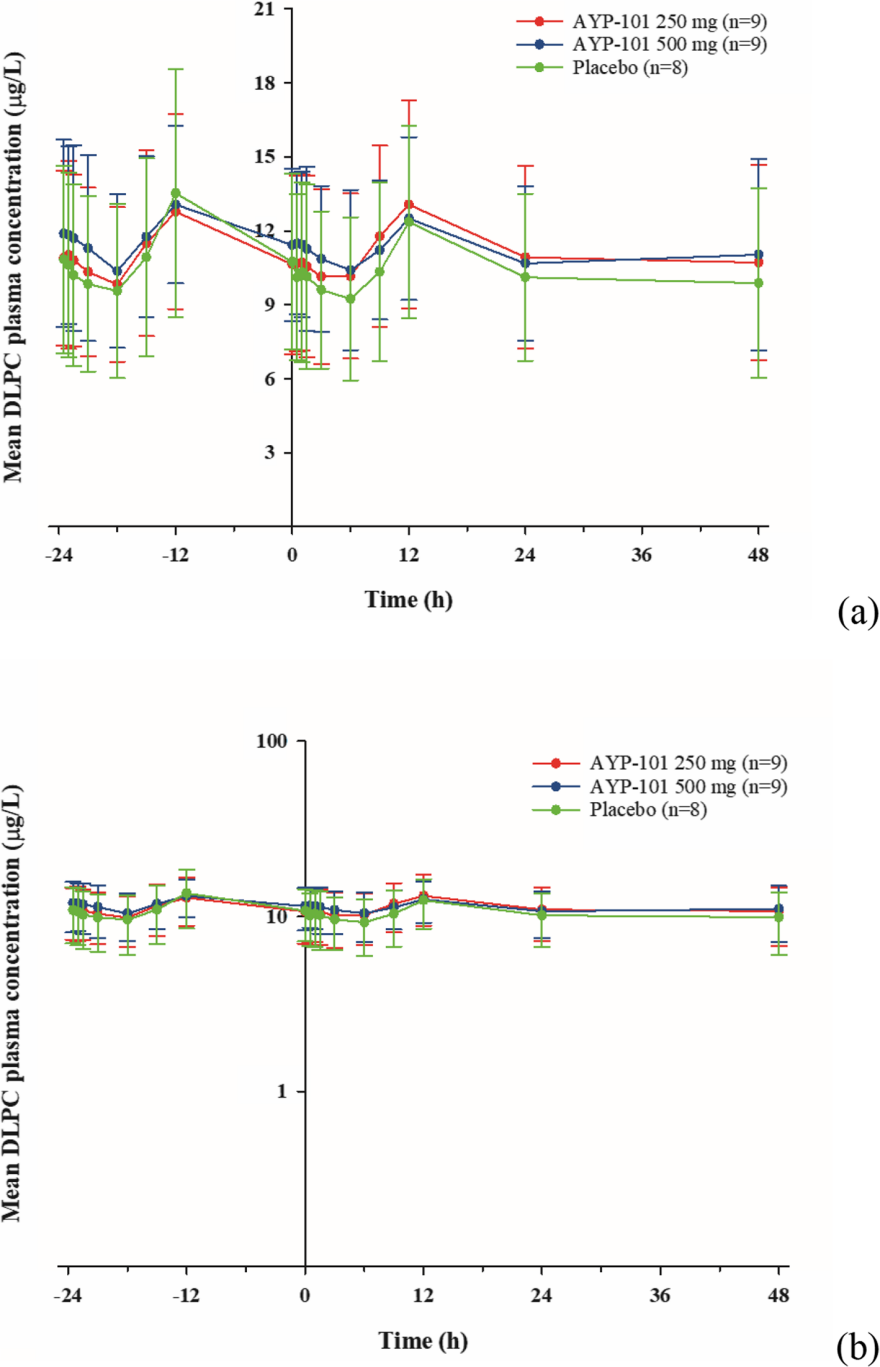
Linear (**a**) and semilogarithmic (**b**) plots of the mean plasma concentration-time profiles of 1,2-dilinoleoyl-sn-glycero-3-phosphocholine (DLPC) following the single administration session of AYP-101 or placebo. The error bars represent the standard deviations

Although DLPC is an endogenous substance with high interindividual variability, DLPC concentrations exhibited similar circadian rhythmic PK profiles among all the dose groups, both before and after a single subcutaneous administration of AYP-101 under the chin in the submental fat (Fig. 2).

**Fig. 3:**
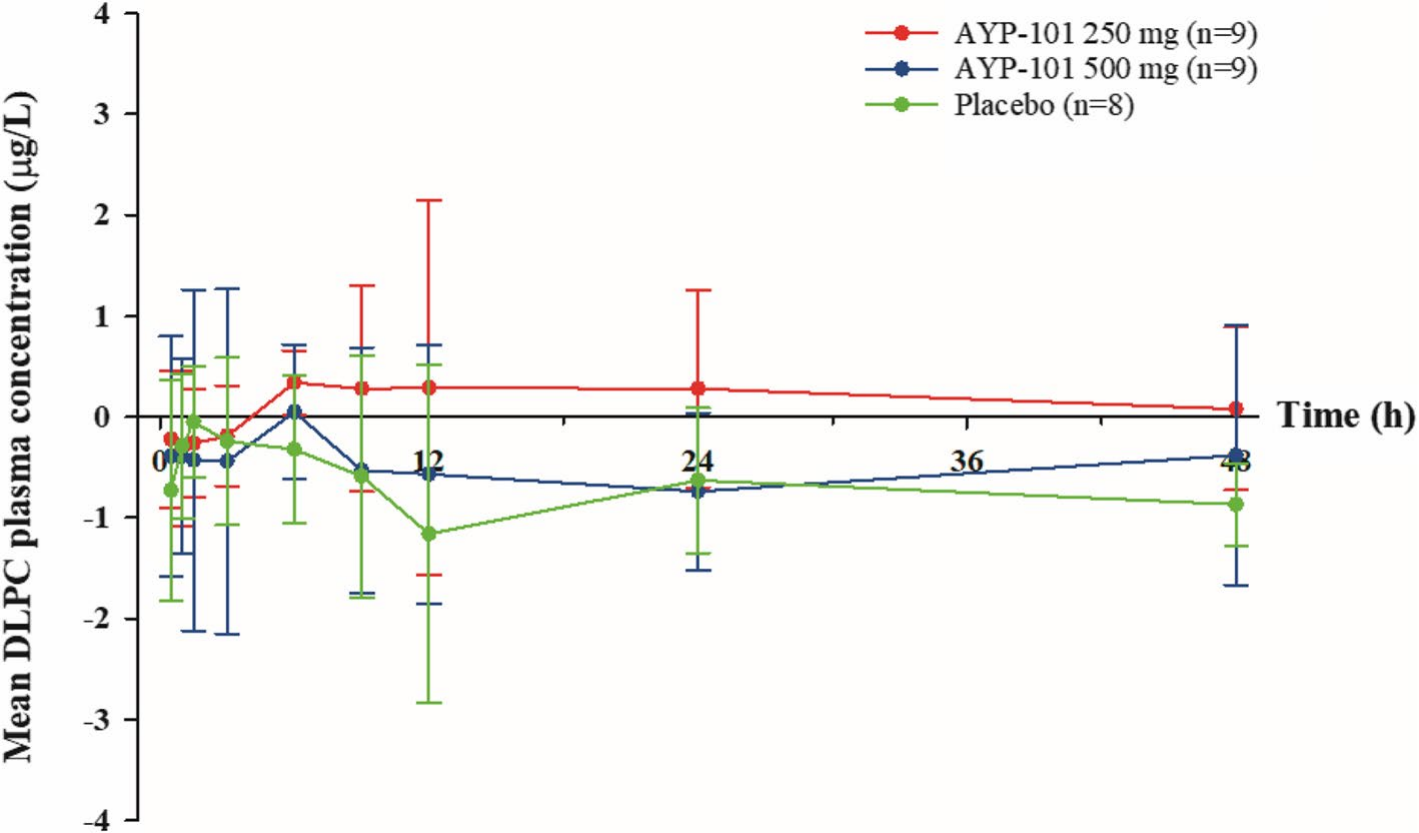
Linear plots of baseline adjusted mean plasma concentration-time profiles following a single administration session of AYP-101 or placebo. The error bars represent the standard deviations. The baseline was adjusted by subtracting the respective contrast baseline concentration from the postdose concentration

The median T_max_ of DLPCs was approximately 12.17 h in both the AYP-101 250 mg and 500 mg groups and the placebo group. No significant differences were observed in the T_max_ compared with the baseline values in the 250 mg and 500 mg groups (Table 2; Figs. 2 and 3).

**Table 2:** Pharmacokinetic parameters of 1,2-Dilinoleoyl-sn-glycero-3-phosphocholine (DLPC) after single Administration Session of AYP-101

Parameter	AYP-101 250 mg	AYP-101 500 mg	Placebo
	*n* = 9	*n* = 9	*n* = 8
T_max_ (h)	12.17 (9.17–12.23)	12.17 (0.67–48.17)	12.17 (12.17–12.17)
C_max_ (µg/L)	13.12 ± 4.19	12.71 ± 3.37	12.36 ± 3.90
ΔC_max_ (µg/L)	0.26 ± 1.74	−0.73 ± 1.46	−1.25 ± 1.51
AUC_0−24_ (µg·h/L)	278.06 ± 90.50	274.79 ± 74.54	258.62 ± 85.30
ΔAUC_0−24_ (µg·h/L)	11.75 ± 21.34	−4.82 ± 15.6	−9.01 ± 19.73

T_max_ is shown as median (minimum-maximum), and other parameters are shown as the mean ± standard deviation

The mean ± standard deviations of ΔC_max_ and ΔAUC_0−24 h_ were calculated with the postdose concentration of DLPC after subtraction of the time-matched pre-dose baseline value

*Abbreviations: T*_max_ time to reach maximum plasma concentration, *C*_max_ maximum plasma concentration, *AUC*_0–24_ area under the plasma concentration-time curve from 0 to 24 h

The mean C_max_ of DLPC ranged from 12 to 13 µg/L across the AYP-101 250 mg, 500 mg, and placebo groups. The ΔC_max_, the baseline-adjusted value, was within ± 1.5 µg/L, which is less than 13% of the C_max_ value. Consequently, no significant difference was observed in comparison with the baseline C_max_ or between the dose groups (Table 2; Fig. 3). Similarly, the mean AUC_0−24 h_ ranged from 250 to 280 µg·h/L. The baseline-adjusted value ΔAUC_0−24 h_ was distributed within ± 11.8 µg·h/L, which is less than 5% of the AUC_0−24 h_ value. However, there was no significant difference compared with the baseline AUC_0−24 h_ or between the dose groups (Table 2; Fig. 3).

### Effects on lipids

The mean serum concentrations of total cholesterol and triglycerides after a single subcutaneous dose of AYP-101 were not significantly different from the baseline values. With respect to the serum free fatty acid concentration, the placebo group exhibited a significant difference from the baseline value (Fig. S1)..

The mean ΔC_max_ and ΔAUC_0-24h_ of total serum cholesterol, triglyceride, and free fatty acid were slightly elevated in every dose group (Table 3). Nevertheless, as the values fell within the range of ±10% and the placebo group presented the greatest difference between baseline and postdose concentrations, these values were deemed to be insignificant.

**Table 3:** Serum Concentration Parameters of Total Cholesterol, triglycerides, and free fatty acid after single Administration Session of AYP-101

Substances	Parameter	AYP-101 250 mg	AYP-101 500 mg	Placebo
		*n* = 9	*n* = 9	*n* = 8
Total Cholesterol	T_max_ (h)	3 (1–48)	1 (0–3)	18 (0–48)
	C_max_ (µg/L)	167 ± 31	194 ± 27	180 ± 13
	ΔC_max_ (µg/L)	-3.8 ± 5.6	-3.3 ± 8.9	2.3 ± 11.9
	AUC_0−24 h_ (µg·h/L)	3750 ± 740	4333 ± 603	4026 ± 327
	ΔAUC_0−24 h_ (µg·h/L)	-45.8 ± 74.3	-50.3 ± 121.0	-8.4 ± 153.5
Triglyceride	T_max_ (h)	9 (0–48)	12 (0.50–48)	9 (0–12)
	C_max_ (µg/L)	136 ± 59	112 ± 29	139 ± 52
	ΔC_max_ (µg/L)	-5.7 ± 11.4	-7.1 ± 11.5	-29.0 ± 33.7
	AUC_0−24 h_ (µg·h/L)	2717 ± 1249	2184 ± 580	2792 ± 1095
	ΔAUC_0−24 h_ (µg·h/L)	-158.7 ± 178.9	-235.8 ± 132.4	-484.8 ± 444.9
Free Fatty Acid	T_max_ (h)	3 (0–48)	3 (0–24)	3 (1–24)
	C_max_ (µg/L)	744 ± 205	643 ± 200	598 ± 194
	ΔC_max_ (µg/L)	98.3 ± 82.2	16.0 ± 122.4	-52.1 ± 65.8
	AUC_0−24 h_ (µg·h/L)	6710 ± 2599	5816 ± 2001	6235 ± 1817
	ΔAUC_0−24 h_ (µg·h/L)	370.7 ± 1607.3	-33.0 ± 1525.5	-54.9 ± 890.9

T_max_ is shown as median (minimum-maximum), and other parameters are shown as the mean ± standard deviation

The mean ± standard deviations of ΔC_max_ and ΔAUC_0−24 h_ were calculated with the post-dose concentration of DLPC after subtraction of the time-matched pre-dose baseline value

*Abbreviations: T*_max_ time to reach maximum plasma concentration, *C*_max_ maximum plasma concentration, *AUC*_0-24_ area under the plasma concentration-time curve from 0 to 24 h

## Discussion

The demand for a reduction in excessive submental fat has increased in recent years. Various approaches have been explored, with some focusing on DCA despite concerns about its adverse reactions. To address these concerns, we focused our attention on SPC dissolved with GCA, which offers a more selective and tolerable alternative. To the best of our knowledge, this is the first study to determine the safety, PK and effects of AYP-101, a treatment for excessive submental fat in humans, on the lipid profile.

With respect to the safety results, all AEs except for one case, in which no causal relationship with the investigational product was found, were solicited ADRs. The most common ADR observed was injection site pain, which is consistent with existing knowledge regarding agents containing essential phospholipids (referred to as SPCs in this study), which are known to induce pain, edema, and various local ADRs in adipocytes [15]. For example, solicited ADRs such as bruising and pain at the injection site have been commonly reported with various conventional drugs that contain DCA and SPC, similar to AYP-101 [16].

From the perspective of severity, all the observed ADRs were mild, and the patients fully recovered without sequelae. Additionally, no other clinically significant abnormalities were observed in this study, indicating that AYP-101 was safe and well tolerated at both 250 mg and 500 mg. Many previous clinical studies of the aforementioned DCA have shown frequent ADRs in terms of safety outcomes, such as edema, bruising, pain, and numbness, similar to those of SPC but with severities ranging from mild to moderate [17]. Notably, less frequent yet severe ADRs, including nerve damage, dysphagia, lymph node damage, alopecia, and ulceration, have also been reported in several clinical trials of DCA [17]. In light of these results, the fact that all the ADRs observed in this study were mild suggests the relatively superior safety profile of SPC.

A trend toward a greater incidence of ADRs was subsequently observed in the AYP-101 treatment group than in the placebo group. However, no clinically significant differences in ADRs were observed between the AYP-101 250 mg and 500 mg dose groups, indicating that the dose difference between 250 mg and 500 mg did not significantly affect the incidence of ADRs. Considering its pharmacological effects for future use, AYP-101 is expected to maintain a sufficiently high level of safety at doses up to 500 mg.

The PK results revealed no clinically significant differences between the AYP-101 250 mg and 500 mg dose groups and the placebo group. In addition, no significant differences were observed when the baseline values were compared with the postdose concentrations of AYP-101. These results indicate that a single subcutaneous administration of AYP-101 at doses of 250 mg and 500 mg resulted in a localized effect within the injection site without a further increase in systemic circulation. In comparison, DCA is known to be rapidly absorbed into the systemic circulation and to increase plasma concentrations [18]. These results are consistent with the safety results of AYP-101, which demonstrated the absence of systemic ADRs.

The administration of AYP-101 did not significantly affect total cholesterol, triglyceride, or free fatty acid levels, with changes within 10%. These results are consistent with the results of previous preclinical studies, including a repeated-dose toxicity test in beagle dogs, where a decrease in total cholesterol was observed within normal ranges. Similarly, the results were comparable to those of a previous clinical trial of the combination of DCA or SPC/DCA, which showed no statistically significant changes in serum lipid levels [18, 19]. These results may indicate that subcutaneous injections of 250 mg and 500 mg of AYP-101 may have acted locally without affecting systemic lipid levels. Previous studies of subcutaneous injection of SPCs at various sites have demonstrated site-specific reductions in fat [20]. In a previous study, 10% of the subjects were nonresponders and low responders who had no or minimal change [20]. Therefore, another possibility is that the subjects in this study may have been less responsive to the SPC.

This study included some participants with relatively normal submental fat conditions; therefore, the results may not be generalizable to patients with excessive submental fat under the chin. The study was also limited by a lack of efficacy assessments.

However, our study demonstrated that AYP-101 has a promising safety profile, with only mild and expected side effects observed at both the 250 mg and 500 mg dosages. These findings may offer advantages over traditional treatments, which are known to increase the risk of severe ADRs such as DCA. The favorable safety profile of AYP-101 underscores the need for future late-phase studies in larger and more diverse populations, including obese patients with substantial submental fat, and various study methods, including imaging technology, for accurately assessing the efficacy of submental fat reduction and the localized effects of AYP-101.

## Conclusions

In conclusion, the results of this study demonstrate the excellent safety and tolerability of the newly developed SPC formulation AYP-101 at doses of 250 mg and 500 mg. Moreover, the administration of AYP-101 via subcutaneous injection under the chin resulted in minimal systemic exposure, further confirming its safety profile. These results highlight the promising potential of AYP-101 as a therapeutic agent and are anticipated to provide valuable insights for future clinicians and patients who are using SPC-like drugs.

## Supplementary Information

Supplementary Material 1.

## Data Availability

No datasets were generated or analysed during the current study.

## Abbreviations

ADR: Adverse drug reaction
AE: Adverse events
AUC_inf_: Area under the plasma concentration‒time curve extrapolated to infinity
AUC_0-24h_: Area under the plasma concentration-time curve up to 24 hours
CL: Clearance
C_max_: Maximum plasma concentration
DCA: Deoxycholic acid
DLPC: 1,2-dilinoleoyl-sn-glycero-3-phosphocholine
ΔC_max_: Baseline adjusted C_max_
ΔAUC_0-24h_: Baseline adjusted AUC_0-24h_
GCA: Glycocholic acid
LC‒MS/MS: Liquid chromatography coupled with tandem mass spectrometry
λ_z_: Elimination rate constant
PK: Pharmacokinetic
SMF: Submental fat
SPC: Soybean phosphatidylcholine
T_max_: Time to reach C_max_
t_1/2_: Terminal half-life
V_d_: Volume of distribution
