# Supplementary material for: Phase 1 study of AYP-101 (soybean phosphatidylcholine): safety, pharmacokinetics, and lipid profile effects for reducing submental fat
Source: Lipids Health Dis. 2024 Dec 28;23:426. doi: 10.1186/s12944-024-02387-4 (PMC11681640; doi:10.1186/s12944-024-02387-4)
Supplement: Supplementary file 1 — Supplementary Material 1. [file 12944_2024_2387_MOESM1_ESM.docx]

**Table S1. Summary of Demographics.**

| **Variable** | **AYP-101 250 mg (N=9)** | **AYP-101 500 mg (N=9)** | **Placebo (N=8)** | **All Subjects (N=26)** | ***p* value** |
| --- | --- | --- | --- | --- | --- |
| Sex | | | | | |
| Male | 7 (77.8) | 8 (88.9) | 7 (87.5) | 22 (84.6) | 1.0000 ^[a]^ |
| Female | 2 (22.2) | 1 (11.1) | 1 (12.5) | 4 (15.4) |  |
| Age (years) | 29.44 ± 7.09 | 26.00 ± 2.24 | 28.75 ± 5.70 | 28.04 ± 5.40 | 0.3971 ^[b]^ |
| Height (cm) | 171.08 ± 9.76 | 176.58 ± 5.89 | 174.34 ± 6.29 | 173.98 ± 7.62 | 0.2932 ^[b]^ |
| Weight (kg) | 77.20 ± 12.05 | 84.39 ± 13.83 | 85.95 ± 12.97 | 82.38 ± 13.04 | 0.3150 ^[b]^ |
| BMI (kg/m^2^) | 26.39 ± 3.77 | 26.99 ± 3.85 | 28.18 ± 3.67 | 27.15 ± 3.69 | 0.7992 ^[b]^ |
| Percentages are based on the subjects within each treatment group.  Sex shown as N (%) and other data shown as the mean ± standard deviation. [a] Fisher’s exact test [b] Kruskal‒Wallis test | | | | | |


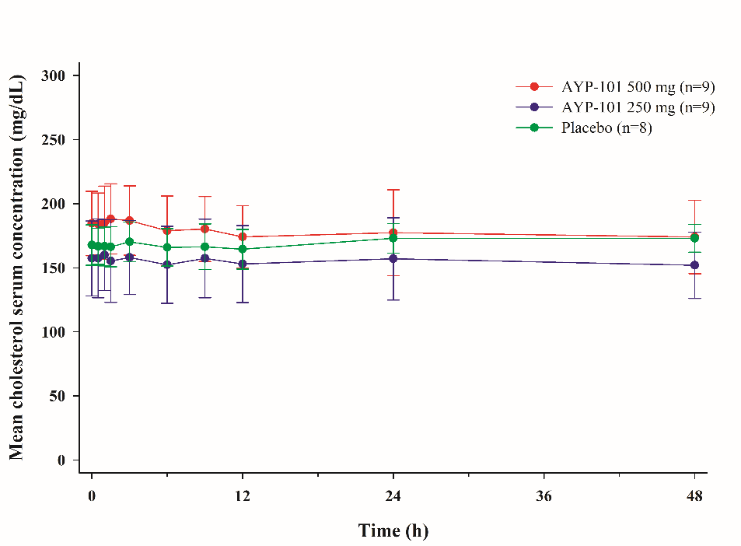


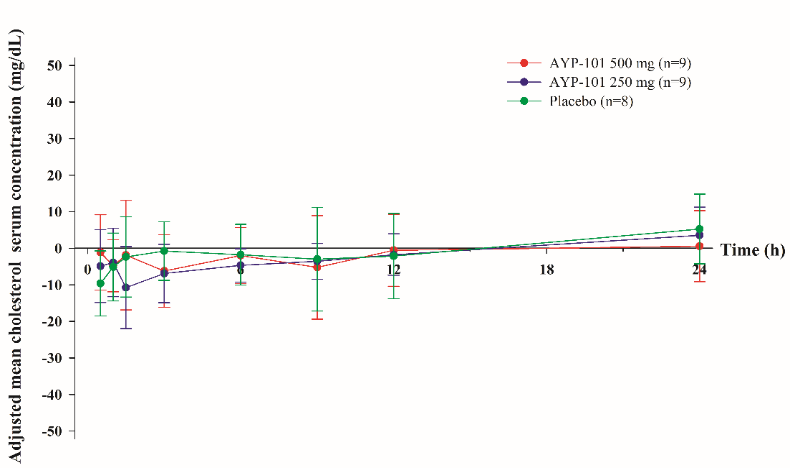


**(a) (b)**


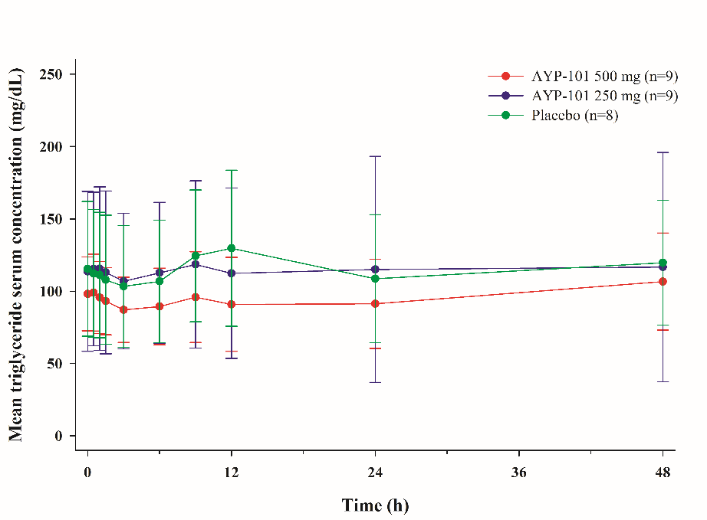


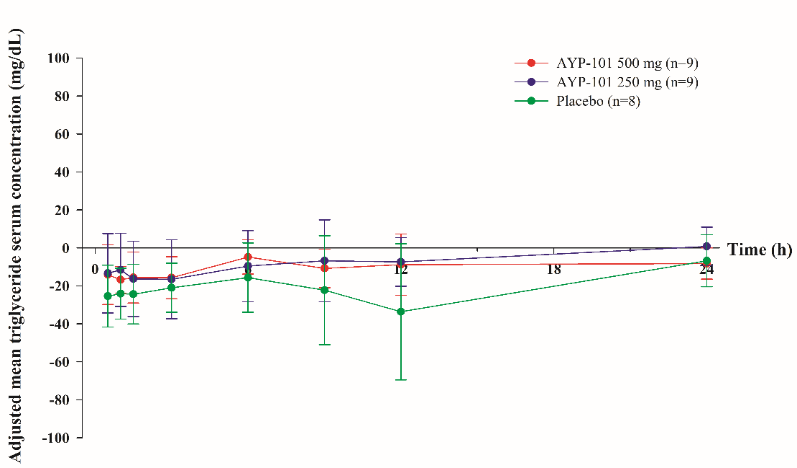


**(c) (d)**


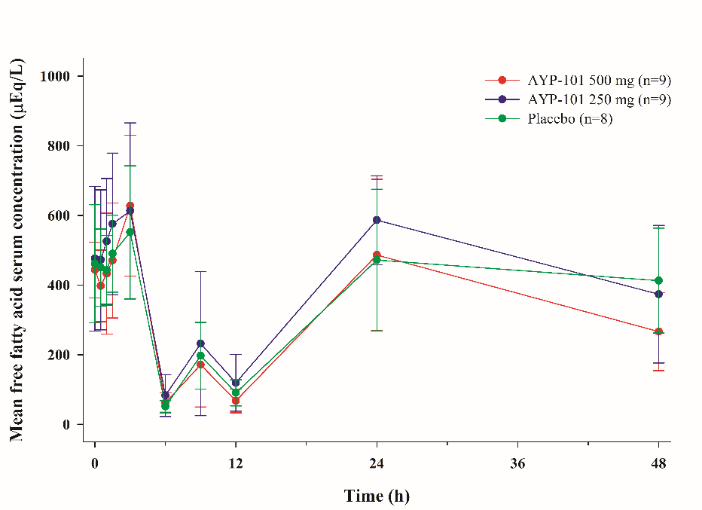


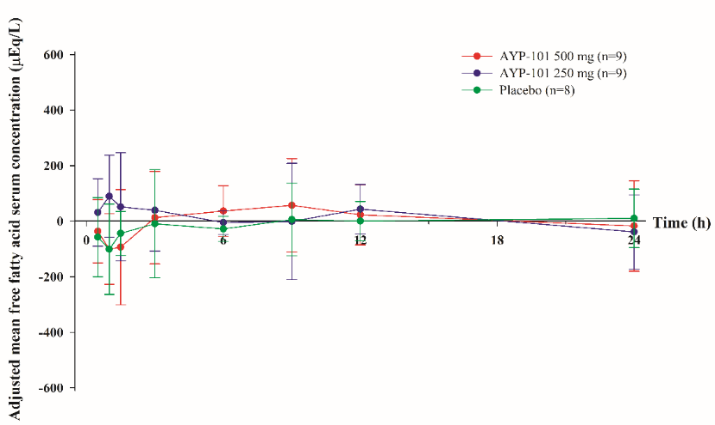


**(e) (f)**

**Figure S1.** Mean serum concentrations - time profiles (a, c, e) and mean baseline adjusted serum concentrations - time profiles (b, d, f) of total cholesterol (a, b), triglycerides (c, d), and free fatty acids (e, f) after a single administration session of AYP-101. The baseline was adjusted by subtracting the respective contrast baseline concentration from the postdose concentration**.**


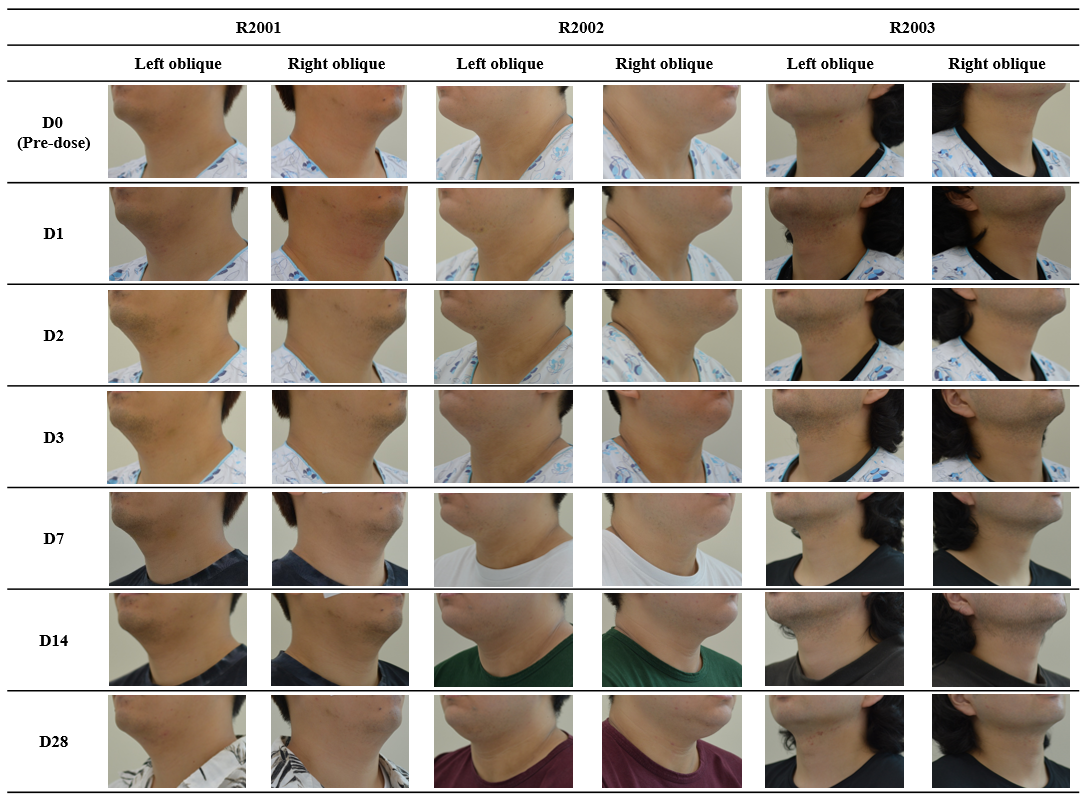


**Figure S2.** Photographs illustrating submental injection sites before (D0) and after the administration of AYP-101 (D1, D2, D3, D7, D14, and D28) in the first three subjects of Cohort **2.**
